# Lineages, Sub-Lineages and Variants of Enterovirus 68 in Recent Outbreaks

**DOI:** 10.1371/journal.pone.0036005

**Published:** 2012-04-20

**Authors:** Ina L. Lauinger, Jon M. Bible, Eugene P. Halligan, Emma J. Aarons, Eithne MacMahon, Cheuk Y. W. Tong

**Affiliations:** 1 Department of Infectious Diseases, School of Medicine, King's College London, London, United Kingdom; 2 Infection Science, GSTS Pathology, London, United Kingdom; 3 Department of Infectious Diseases, Guy's and St. Thomas' NHS Foundation Trust, London, United Kingdom; Hannover Medical School, Germany

## Abstract

Enterovirus 68 (EV68) was first isolated in 1962. Very few cases of EV68 infection were described over the ensuing 40 years. However, in the past few years, an increase in severe respiratory tract infections associated with EV68 has been reported. We identified two clusters of EV68 infection in South London, UK, one each in the autumn/winters of 2009 and 2010. Sequence comparison showed significant homology of the UK strains with those from other countries including the Netherlands, Japan and the Philippines, which reported EV68 outbreaks between 2008 and 2010. Phylogenetic analysis of all available VP1 sequences indicated the presence of two modern EV68 lineages. The 2010 UK strains belonged to lineage 2. Lineage 1 could be further divided into two sub-lineages: some Japanese and Dutch strains collected between 2004 and 2010 form a distinct sub-lineages (sub-lineage 1.1), whereas other strains from the UK, Japan, Netherlands and Philippines collected between 2008 and 2010 represent sub-lineage 1.2. The UK 2009 strains together with several Dutch and Japanese strains from 2009/2010 represents one variant (1.2.1), whereas those from the Philippines a second variant (1.2.2). Based on specific deletions and substitutions, we suggest rules for the assignment of lineages and sub-lineages. Molecular epidemiological analysis indicates rapid recent evolution of EV68 and this may explain the recent findings of a global resurgence of EV68. Continuous global monitoring of the clinical and molecular epidemiology of EV68 is recommended.

## Introduction

Enterovirus 68 (EV68) is a unique virus in the enterovirus genus as it shares characteristics with rhinoviruses, such as infection of the respiratory tract and acid lability [1]. EV68 does not follow the usual summer-autumn seasonality observed for most other enterovirus species [2], although a recent outbreak in the Netherlands occurred in the autumn of 2010 between September and November [3]. It was first isolated from throat swabs collected from four hospitalised children in 1962 in the USA [4] and together with EV70 and EV94, was classified as enterovirus species D. Rhinovirus 87 was found to be identical to EV68 and reclassified into enterovirus species D [1], [5], [6]. Apart from respiratory tract infections, EV68 has also been implicated in some rare cases of fatal central nervous system infection [2], [7]. An American surveillance system reported the most common age group affected by EV68 to be children aged between 1 and 4 years, but noted that about a quarter of all infections were reported in adults [2]. A more recent study found >50% of all EV68 infections occurred in adults over the age of 40 [3].

Since its initial discovery in the 1960s, EV68 infection was only reported sporadically in the literature. Enterovirus surveillance in the USA between 1970 and 2005 showed that EV68 was one of the most rarely reported serotype with 26 reports observed over 36 years of surveillance in USA [2]. However, in the last few years between 2008 and 2010, an increasing number of clusters of acute respiratory illness associated with EV68 were reported in Asia, Europe, and the USA [8], [9], [10], [11], [12], [13]. Some recent observations of increase in cases could be partly due to enhanced surveillance in patients with asthma [1]. However, a review of recent EV68 reports suggested that the increase may not be entirely accounted for by reporting bias, as reports have come independently from countries in different continents over the same period and some countries, like USA and Japan, have been conducting continuous enterovirus surveillance for many years. A retrospective study in the Netherlands using samples dating back to 1996 confirmed the re-emergence of EV68 [3]. In many cases, EV68 was the sole enterovirus serotype detected and was associated with severe or even fatal disease (Table S1).

Two clusters of EV68 infection were noted in our centre in South London between November 2009 and December 2010. The first cluster occurred during the winter of 2009 and the second in the autumn/winter of 2010. We performed a molecular epidemiological investigation of these EV68 strains and compared our findings to that of other recent viral sequences reported from other countries.

## Materials and Methods

### Patients

As part of the work up during pandemic influenza seasons, respiratory samples were obtained from patients with respiratory symptoms attending primary care or Guy's and St. Thomas' Hospitals in London, UK. Samples submitted to the diagnostic laboratory between November 2009 and December 2010 were investigated for viral pathogens using a multiplex nucleic acid amplification panel (ResPlex II v2, Qiagen or xTAG RVP FAST v1, Luminex). The nucleic acid targets of the multiplex panel consisted of influenzaviruses A and B, parainfluenzaviruses 1–4, respiratory syncytial viruses A and B, human metapneumovirus, adenoviruses, coronaviruses, bocavirus and entero/rhinoviruses. Respiratory specimens used include nasopharyngeal aspirate, nasal swab, throat swab and bronchoalveolar lavage. Samples that tested positive for entero/rhinovirus RNA were further analysed by direct sequencing into individual enterovirus and rhinovirus subtypes. This study was considered by the Chairman of the Research Ethics Committee of St. Thomas' Hospital and was advised that ethical review of this study was not required under the terms of the Governance Arrangements for Research Ethics Committees in the UK (St Thomas' Research Ethics Committee Reference: 10/08). The demographics of EV68 positive patients and patients positive for other entero/rhinoviruses were compared by descriptive analyses. Pearson's chi square test, Fisher's exact test or one-way ANOVA were used for analysis and p-values<0.05 were considered significant. All analyses were performed in SPSS Statistics 19.

### Molecular Analysis

RNA was extracted from the clinical specimens using the QIAamp Viral RNA Mini Kit (Qiagen) according to the manufacturer's instructions. cDNA was synthesised using random primers (Invitrogen), M-MLV Reverse Transcriptase (Invitrogen) and RNaseOut (Invitrogen) with 10 min at 25°C, 60 min at 37°C and 5 min at 94°C. Samples were screened by PCR with the HotStarTaq Master Mix Kit (Qiagen) targeting the 5′NTR using primers DK001 [14] and DK004 [15] under the following conditions: 15 min at 95°C, 30 sec at 94°C, 30 sec at 53.4°C, 30 sec at 72°C (45 cycles) and 10 min at 72°C. Amplification of the VP4/VP2 region was performed as above with primers VP4/2 F and VP4/2 R [16] or RCV556F (ACT ACT TTG GGT GTC CGT GTT TC) and RCV886R (TTT CCR ATA GTG ATT TGC TTK AGC C) with 60°C annealing and 40 cycles or 52°C and 40 cycles, respectively. VP1 amplification was performed using primers EV68-VP1-2325-fwn and EV68-VP1-3121-rvni or EV68-VP1-2547-fwni and EV68-VP1-2772-rvni2 [13] with 55°C annealing temperature and 40 cycles.

Bidirectional sequencing was performed by LGC Genomics GmbH (Berlin, Germany) or in house (PCR product cleaning with microClean (Microzone), cycle sequencing with BigDye Terminator v3.1 Cycle Sequencing Kit (Applied Biosystems) on the automated sequencer 3130xl Genetic Analyzer (Applied Biosystems)).

### Sequence Analysis

Sequence analysis was performed using the software BioEdit (version 7.0.9) and MEGA5 (version 5.03). Phylogenetic trees were generated using the neighbour-joining method with Kimura 2-parameter as substitution model and pairwise deletion for gaps and missing data. The Bootstrap method was chosen for the phylogenetic test with 1000 replications. All available GenBank entries submitted for 5′NTR (n = 46), VP4 (n = 93), VP1 (n = 201) and full genomes or coding sequences (n = 6) for EV68 were included in the analysis (Table S2). Sequences from this study were submitted to GenBank with the Accession numbers JQ586203–JQ586249.

## Results

Of the 473 entero/rhinovirus positive respiratory specimens collected between November 2009 and December 2010, a total of 403 were available for this study. Of these, 342 (85%) could be sequenced, of which 36 enteroviruses (10.5%) were identified (n = 22 by 5′NTR and VP4/VP2, n = 12 by 5′NTR only and n = 2 by VP4/VP2 only). Half of these enteroviruses were identified as EV68 (18 specimens from 17 patients). The EV68 specimens were collected in two separate clusters – the first cluster occurred between November and December of 2009 (n = 9) and a second cluster between September and December 2010 (n = 8). No EV68 was detected in between the two winter seasons. The patient age ranged from 7 weeks to 45 years (mean 12.2 years, median 5.8 years) with a male∶female ratio of 10∶7. Apart from one specimen from primary care, the majority of patients were hospitalised (n = 16) with 8 patients requiring intensive care. A co-pathogen was detected in 2 patients with severe illness, one HIV infected patient with cytomegalovirus, and another with *Neisseria meningitidis* septicaemia. Most patients (n = 14) had underlying medical conditions, the majority being chronic respiratory illnesses (n = 9, p = 0.017). The HIV infected patient died. EV68 infection was also significantly associated with airway diseases such as asthma (p = 0.025) (Table 1).

**Table 1:** Clinical and demographic details of EV68 positive and other entero/rhinovirus positive patients.

	EV68 positive patients (%)	Other positive patients (%)	OR (95% CI)	p- value
Total	17	254	na	na
Age (years) (mean, median)	10.2, 5.1	12.4, 1.4	na	0.669
Male	10 (58.8%)	148 (58.3%)	0.977 (0.360–2.650)	0.964
Hospital admission	16 (94.1%)	240 (94.5%)	1.071 (0.132–8.670)	1
Admitted from Accident & Emergency Department	8 (47.1%)	84 (33.1%)	0.556 (0.207–1.492)	0.238
Lengths of stay (days) (mean, median)	6.6, 3	18.3, 5	na	0.316
Incidental finding	1 (6.3%)	50 (19.7%)	3.676 (0.474–28.494)	0.321
Mild infection	7 (43.8%)	103 (40.6%)	0.877 (0.317–2.430)	0.801
Severe infection^1^	8 (50%)	101 (39.8%)	0.660 (0.240–1.816)	0.418
Underlying risk factors	13 (81.3%)	185 (72.8%)	0.619 (0.171–2.238)	0.571
Death	1 (6.3%)	17 (6.7%)	1.081 (0.135–8.677)	1
Chronic respiratory diseases	9 (56.3%)	65 (25.6%)	0.267 (0.096–0.747)	**0.017**
Chronic heart diseases	3 (18.8%)	45 (17.7%)	0.933 (0.255–3.410)	1
Chronic neurological diseases	1 (6.3%)	28 (11%)	1.858 (0.236–14.610)	1
Immunosupression	3 (18.8%)	40 (15.7%)	0.810 (0.221–2.972)	0.726
Other risk factors	3 (18.8%)	70 (27.6%)	1.649 (0.456–5.960)	0.570
Upper respiratory tract infection	3 (18.8%)	104 (40.9%)	3.004 (0.835–10.806)	0.078
Airway disease (e.g. asthma)	7 (43.8%)	48 (18.9%)	0.300 (0.106–0.845)	**0.025**
Lower respiratory tract infection	6 (37.5%)	102 (40.2%)	1.118 (0.394–3.173)	0.833
Coinfection	2 (12.5%)	63 (24.8%)	2.309 (0.511–10.438)	0.372

1 An infections was classified as severe if the patient required intensive care, attended a high dependency unit or died.

### Nucleotide and phylogenetic analysis

The RNA sequences of EV68 detected in 2010 were different from that of 2009. The only exception was strain 09-70 which came from a sample from December 2009, but its 5′NTR and VP1 sequences clustered with the 2010 EV68 strains. The overall nucleotide similarity within each cluster were at least 99.7% and 99.1% in the 5′NTR, 98.9% and 97.7% in the VP4/VP2, and 98.5% and 97.5% in the VP1 region for the 2009 and 2010 (including the strain 09-70) respectively.

#### 5′NTR

**Figure 1:**
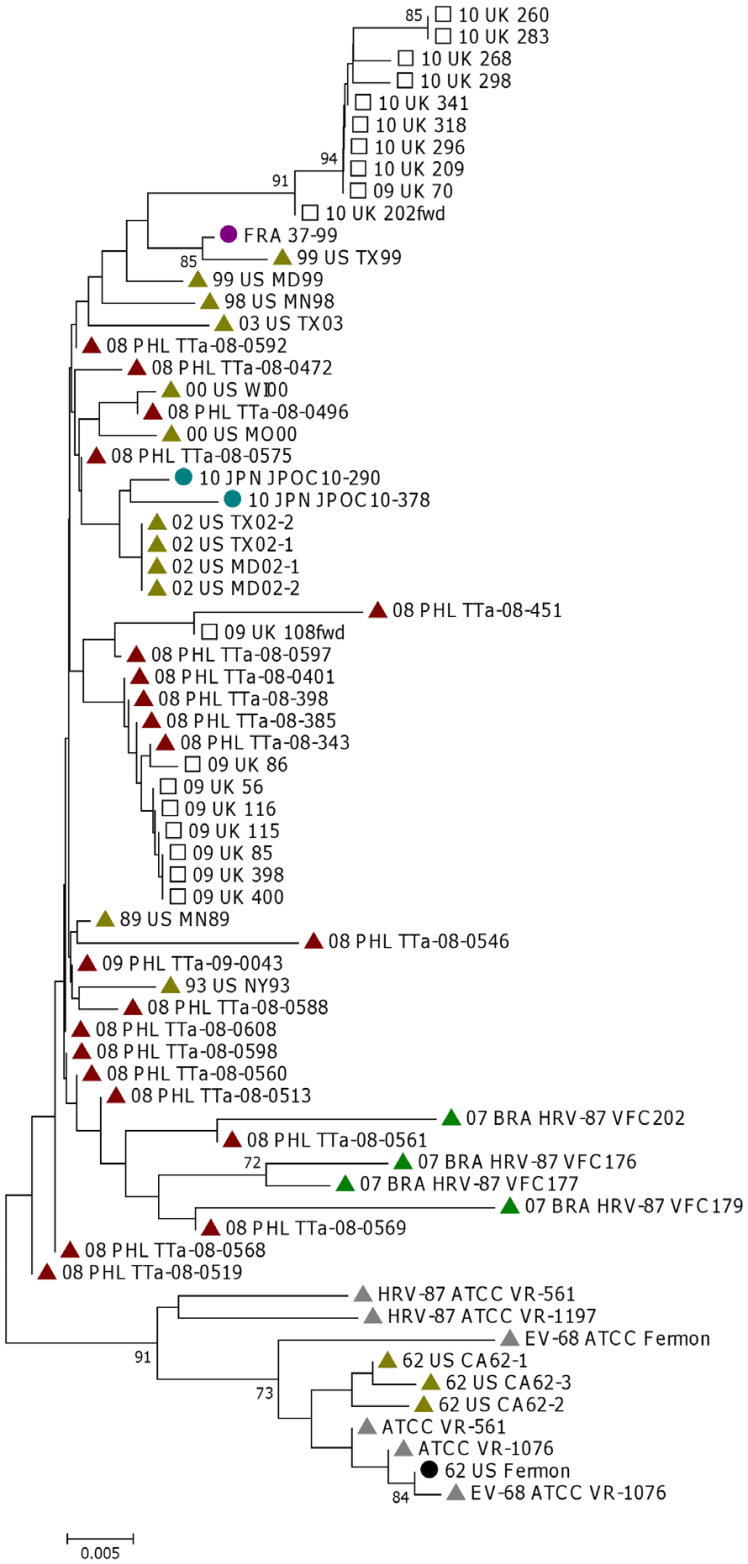
Phylogenetic analysis of 5′NTR. First two letters of sequence are collection year, followed by letters for the country of origin.

**Figure 2:**
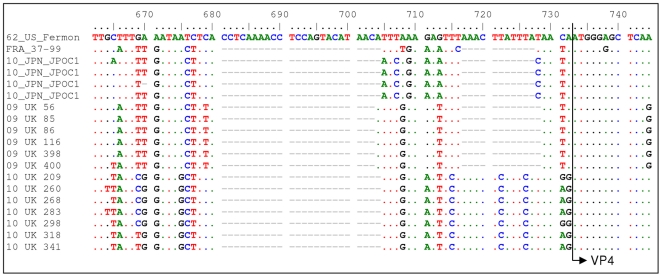
5′NTR sequences preceding VP4 showing the two deletion blocks.

As expected, the conserved 5′NTR showed a high degree of similarity between the two clusters (>95.8%). They both showed a high degree of homology to the prototype Fermon strain from 1962 (>95.3%) and the full length French strain 37–99 from 1998 (>97.5%). However, the 2010 5′NTR sequences showed more divergence in comparison to recent Filipino sequences from 2008/2009 (>97.4% for 2009 sequences versus >95.3% for 2010). Phylogenetic analysis of this region showed close clustering of the 2009 UK strains with 6 other Filipino strains from 2008, whereas the 2010 UK strains together with the 2009 09-70 strains form a separate cluster (Figure 1). Comparing the sequence from 2009 and 2010 showed the presence of eight nucleotide variations between the two years in the 5′NTR (Table 2). Several of these mutations are reported for the first time (T318C and C319T in 2010, and A495G in 2009 sequences, using the Fermon strain as reference). In addition, two blocks of deletions (681–704 and 717–727) were noted in the 5′NTR among the more recently reported Japanese isolates from the literature compared to the Fermon strain [11]. Our 2009 and 2010 strains also had these deletions although the islet region between the two deletion blocks (705–716) differed in at least 5 out of 12 nucleotides compared to the Japanese strains (Figure 2). Additionally, whereas the 2009 UK strains had both deletion blocks (681–703 and 717–728), the 2010 strains (including 09-70) only had the deletion between 681–704. Whether or not these deletion blocks occurred in other EV68 strains is not known, as other EV68 5′NTR sequences reported in the GenBank were not extended to cover this pre-VP4 region of the 5′NTR.

**Table 2:** Sequence variations in 5′NTR between UK 2009 and 2010 EV68 sequences.

Position^1^	Mutation^1^	Found in	Also found in
222*	T→C	2009 (except 09-116)	All Japan (2010), 11/15 US (1989–2003), 1/4 Brazil (2007)
279	T→C	2009	4/21 Philippine (2008/09)
318+319	T→C+C→T	2010	None
333	G→A	2009	All Japan (2010), all Philippine (2008/09), 10/15 US (1989–2003), 1/4 Brazil (2007)
345	C→T	2010	All Brazil (2007), 1/21 Philippine (2008/09), 1/15 US (2003)
471*	C→T	2010	All Japan (2010), France (1998), all Brazil (2007), 11/15 US (1989–2003)
495*	A→G	2009	None
518*	C→T	2009 (and 10-202)	All Japan (2010), France (1998), 13/15 US (1962–2003)

1 reference Fermon strain (AY426531).

* Filipino sequences not amplified into this region.

#### VP4/VP2

**Figure 3:**
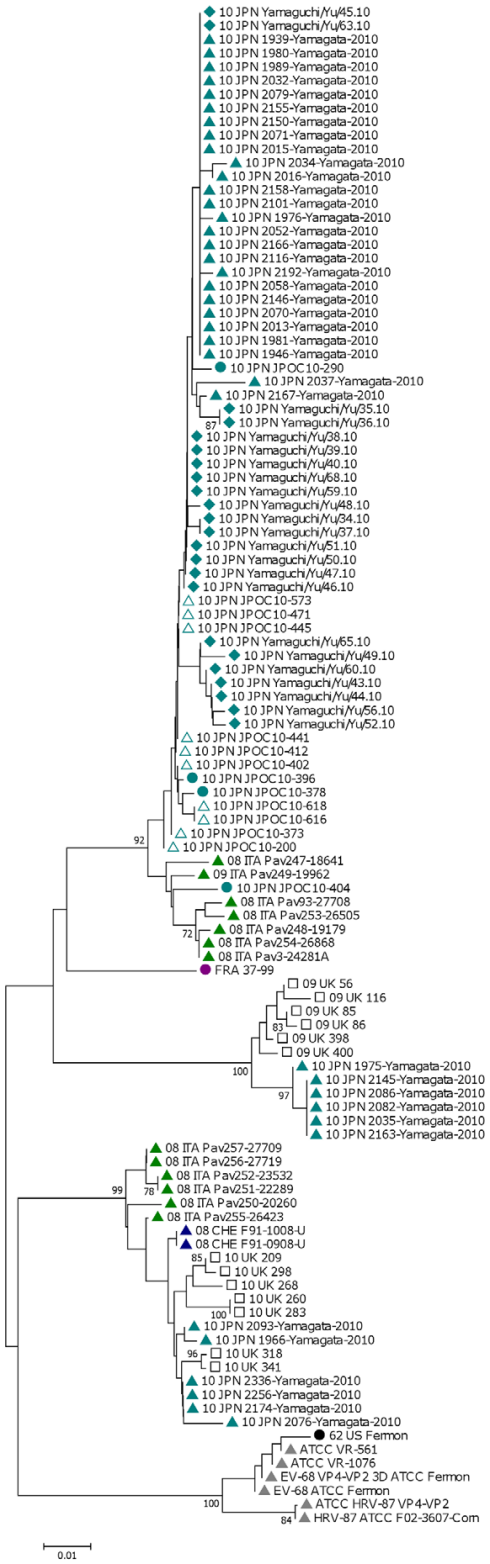
Phylogenetic analysis of VP4/VP2. First two letters of sequences are collection year, followed by letters for the country of origin.

**Figure 4:**
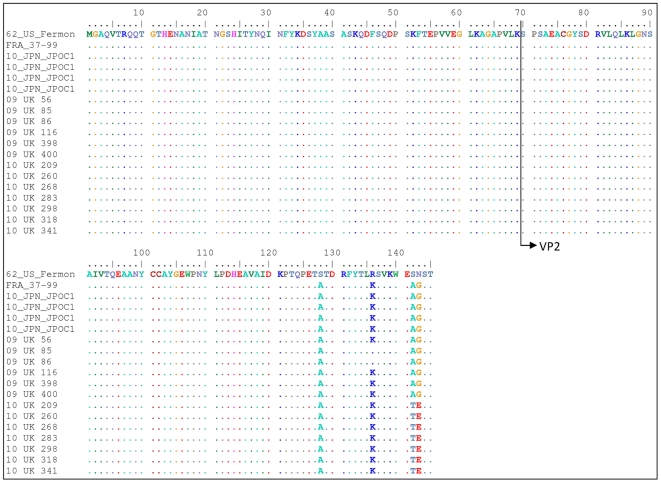
Protein sequence for VP4/VP2 of all available sequences.

The VP4/VP2 region showed a much greater difference between the two clusters (87.4%–89.7% nucleotide similarities) but retained a high conservation in the protein (97.9%–98.6% amino acid similarities). The UK 2009 sequences had a high degree of homology to 6 other Japanese sequences from 2010 (Figure 3). For example, the Yamagata 2035 strain from 2010 was 98.4% similar to the UK 09-398 strain, but only 89.2% similar to the UK 10-268 strain. On the other hand, the UK 2010 sequences were more similar to another cluster of Japanese strains from the same year (Figure 3). For example, the Yamagata 2076 strain also from 2010 was 98.1% similar to the UK 10-268 strain, but only 90.0% similar to the UK 09-398 strain. The UK 2010 sequences also showed a high degree of homology to some older European sequences. The 2008 Swiss F91-1008-U strain showed 98.6% and the 2008 Italian Pav250-20260 strain 98.0% similarity to UK 10-268 strain. The corresponding homology to the UK 09-398 strain was 90.7% and 91.0% respectively. Overall, the amino acid composition in the VP4 protein (1–69) showed a high degree of conservation (only change was I19V in Yamaguchi/Yu/50.10). In the VP2 region, there were only four amino acid changes compared to the Fermon strain (Figure 4). Interestingly, whereas UK 2009 strains showed the same substitutions as the 1998 French and 2010 Japanese JPOC strains (S142A and N143G), the UK 2010 isolates have different substitutions at these positions (S142T and N143E). All other available sequences were not extended to cover this region.

#### VP1

VP1 is the region in which the largest number of sequences of previous isolates was available from the GenBank. The homologies between the 2009 and 2010 (including 09-70) UK sequences in this region were between 86.3%–90.7% (nucleotides, Table 3) and 91.0%–95.0% (amino acids, Table 4). Distinct clustering of sequences from the two years was again clearly observed (Figure S1, S2, S3). The UK 2009 sequences were most similar to 7 Japanese strains from 2010 (same as for VP4/VP2) and 14 Dutch strains from 2009/2010. The 2010 UK sequences (including 09-70), on the other hand, were most similar to a number of other Dutch strains from 2006–2010 and 6 other Japanese strains from 2010 (same as for VP4/VP2). No VP1 sequences from the older Swiss and Italian strains from 2008 were available for comparison. Both the 2009 and 2010 UK VP1 sequences diverged significantly from the prototype Fermon strain (maximal homology of 88.1% and 89.3% respectively), but were closer to another earlier full genome sequence from France (strain 37–99 from 1998 – 93.5% and 91.8% maximal homology respectively). All Filipino sequences from 2008/2009, including the ones which clustered closely with the UK 2009 sequences at the 5′NTR, form a distinct cluster at the VP1 region, the separate branching of which was supported by a bootstrap value of 98 (Figure S2). A three nucleotide deletion (ATG), resulting in the deletion of glycine at position 141 in the VP1 protein (Figure S4) [13], was noted for all UK 2010 strains and all strains clustering closely, including strains from the Netherlands (2001–2010), USA (2003), Finland (2004/2005) and Japan (2006–2010).

**Table 3:** Nucleotide identities in the VP1 region for clusters and lineages.

		within		UK 2009		UK 2010	
	n	MIN	MAX	MIN	MAX	MIN	MAX
**Variant 1.2.1**	**27**	**97.4%**	**100.0%**				
UK 2009	6	98.5%	100.0%	98.5%	100.0%	86.3%	90.7%
Netherlands 2009–2010	10	97.8%	100.0%	98.0%	100.0%	87.3%	90.9%
Netherlands 2009*	4	98.5%	99.7%	97.9%	100.0%	87.3%	89.7%
Japan Yamagata 2010	7	99.2%	100.0%	98.3%	99.5%	87.3%	90.6%
**Variant 1.2.2**	**16**	**97.6%**	**100.0%**				
Philippines 2008	16	97.6%	100.0%	96.1%	98.1%	83.7%	92.3%
**Sub-lineage 1.1**	**70**	**94.7%**	**100.0%**				
Japan JPOC CG/cds^1^ 2010	4	98.5%	99.6%	89.2%	93.8%	84.8%	90.6%
Japan Yamagata 2005–2007	11	98.9%	100.0%	91.7%	94.8%	86.3%	91.3%
Netherlands 2004–2009	18	94.7%	100.0%	90.3%	92.6%	85.3%	90.2%
Japan JPOC 2010	10	98.6%	100.0%	90.2%	93.8%	84.8%	90.3%
Japan Yamagata 2010	27	98.8%	100.0%	89.7%	93.5%	85.3%	90.3%
**Lineage 2**	**68**	**96.1%**	**100.0%**				
UK 2010	10	97.5%	100.0%	86.3%	90.7%	97.5%	100.0%
Netherlands 2010	16	97.7%	100.0%	88.2%	90.9%	96.6%	100.0%
Netherlands 2006–2010*	11	96.7%	100.0%	86.9%	89.7%	96.1%	99.5%
Netherlands 2009–2010*	23	98.5%	100.0%	86.8%	90.2%	97.3%	100.0%
Japan Yamagata 2010	6	98.6%	100.0%	87.4%	90.4%	97.6%	100.0%

1 CG: complete genome, cds: coding sequence.

**Table 4:** Amino acid identities in the VP1 region for clusters and lineages.

		within		UK 2009		UK 2010	
	n	MIN	MAX	MIN	MAX	MIN	MAX
**Variant 1.2.1**	**27**	**97.2%**	**100.0%**				
UK 2009	6	98.5%	100.0%	98.5%	100.0%	91.0%	95.0%
Netherlands 2009–2010	10	97.6%	100.0%	98.0%	100.0%	91.0%	95.5%
Netherlands 2009*	4	98.2%	100.0%	97.3%	100.0%	91.1%	94.0%
Japan Yamagata 2010	7	99.0%	100.0%	98.5%	100.0%	92.5%	94.5%
**Variant 1.2.2**	**16**	**96.9%**	**100.0%**				
Philippines 2008	16	96.9%	100.0%	97.6%	100.0%	88.1%	95.3%
**Sub-lineage 1.1**	**70**	**97.3%**	**100.0%**				
Japan JPOC CG/cds^1^ 2010	4	99.5%	99.8%	95.0%	97.0%	92.9%	97.0%
Japan Yamagata 2005–2007	11	99.7%	100.0%	96.0%	97.0%	93.9%	97.0%
Netherlands 2004–2009	18	97.3%	100.0%	94.7%	97.0%	92.0%	98.5%
Japan JPOC 2010	10	99.0%	100.0%	95.0%	97.0%	92.9%	97.0%
Japan Yamagata 2010	27	99.0%	100.0%	95.5%	97.0%	93.4%	97.0%
**Lineage 2**	**68**	**97.0%**	**100.0%**				
UK 2010	10	98.5%	100.0%	91.0%	95.0%	98.5%	100.0%
Netherlands 2010	16	98.8%	100.0%	92.5%	95.5%	97.0%	100.0%
Netherlands 2006–2010*	11	97.3%	100.0%	89.3%	95.5%	97.0%	100.0%
Netherlands 2009–2010*	23	98.9%	100.0%	88.9%	94.0%	98.4%	100.0%
Japan Yamagata 2010	6	100.0%	100.0%	92.9%	95.5%	97.6%	100.0%

1 CG: complete genome, cds: coding sequence.

### Lineages

**Figure 5:**
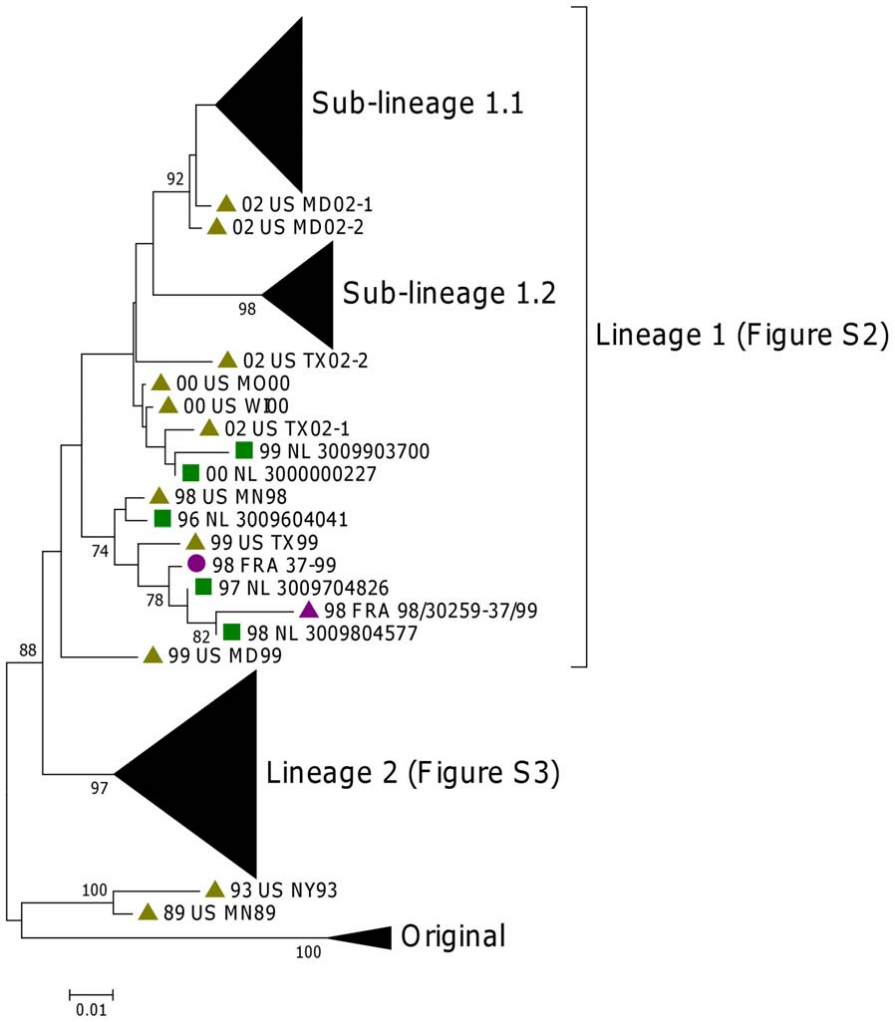
Phylogenetic analysis of VP1. First two letters of sequences are collection year, followed by letters for the country of origin. The subtrees of sub-lineages 1.1 and 1.2 and lineage 2 were collapsed for easy viewing. Figure S1 shows the uncollapsed version. Full details on each lineage can be viewed in Figure S2 and S3.

Phylogenetic analysis of all available VP1 sequences showed that all modern EV68 strains were derived from two separate lineages, supported by a bootstrap value of 88 (Figure 5).

The 2010 UK EV68 strains (including 09-70) share the same lineage as some of the 2006–2010 Dutch and 2010 Japanese strains. This we will refer to as lineage 2. Characteristic for this lineage is the glycine deletion in position 141 in the VP1 protein. Additionally, the amino acid change at position 142 and 143 to threonine and glutamic acid in the VP2 protein could also be linked to this lineage. The presence of the one deletion block (681–704) in the 5′NTR is also a characteristic feature. Additional sequences covering this region are required for verification; however 2010 isolates from Yamagata which clustered within lineage 2 have limited sequence data available which suggest the absence of the second deletion block (717–727) (Figure S5).

Lineage 1, on the other hand, can be divided into further sub-lineages. One branch of this lineage consisted of a number of Dutch strains (2004–2009) and Japanese isolates from Yamagata and Osaka (2005–2010) (sub-lineage 1.1). The UK 2009 EV68 strains also share the same lineage with some of the 2009/2010 Dutch and Japanese strains (variant 1.2.1), but are distinct from another branch consisting of the 2008/2009 Filipino isolates (variant 1.2.2). All these branches were supported by a strong bootstrap value >92. The nucleotide and amino acid homologies between the lineages, sub-lineages and variants were shown in Tables 5 and 6. The two variants showed 96%–98% nucleotide similarities with each other, compared to 89%–95% with sub-lineage 1.1 and 84%–93% with lineage 2. Similarly, sub-lineage 1.1 shared 85%–92% with lineage 2. Within each lineage, the similarity was between 95%–100%. Whereas in the UK, the two lineages predominated in different years (lineage 1.2.1 in 2009 and lineage 2 in 2010), in other countries such as the Netherlands and Japan, different lineages and sub-lineages co-circulated within the same year.

**Table 5:** Nucleotide identities in the VP1 region for lineages.

	Variant 1.2.1		Variant 1.2.2		Sub-lineage 1.1		Lineage 2	
	MIN	MAX	MIN	MAX	MIN	MAX	MIN	MAX
Original	80.9%	89.6%	79.8%	87.2%	83.5%	88.3%	83.1%	89.3%
Variant 1.2.1	97.4%	100.0%	95.6%	98.1%	89.2%	94.8%	86.3%	91.1%
Variant 1.2.2			97.6%	100.0%	89.7%	94.9%	83.7%	92.9%
Sub-lineage 1.1					94.7%	100.0%	84.8%	92.2%
Lineage 2							96.1%	100.0%

**Table 6:** Amino acid identities in the VP1 region for lineages.

	Variant 1.2.1		Variant 1.2.2		Sub-lineage 1.1		Lineage 2	
	MIN	MAX	MIN	MAX	MIN	MAX	MIN	MAX
Original	86.6%	93.0%	85.2%	90.8%	88.6%	96.1%	86.6%	92.9%
Variant 1.2.1	97.2%	100.0%	94.9%	100.0%	93.9%	97.0%	88.9%	95.9%
Variant 1.2.2			96.9%	100.0%	93.1%	96.9%	88.1%	95.9%
Sub-lineage 1.1					97.3%	100.0%	91.1%	98.5%
Lineage 2							97.0%	100.0%

## Discussion

The epidemiology of EV68 is intriguing. It was first described in 1962 [4] but in the ensuing 40 years, it was reported only sporadically despite regular enterovirus surveillance by public health bodies [2]. Over the last decade, and particularly in the last few years, the number of reports of EV68 increased dramatically and in some cases severe respiratory tract infections [3], [8], [9], [11], [13] and even central nervous system infections [2], [7] were reported. An increase in detection rate may be due to improved testing methods or an increase in multiplex molecular testing of respiratory samples as a result of enhanced respiratory virus surveillance. However, it is not possible to discount the possibility that EV68 is an emerging pathogen. A recent study retrospectively investigated the molecular evolution of EV68 in the VP1 region with time [3] and showed that the original Fermon strain and clusters of selected older sequences (1989–2002) disappeared over time with subsequent emergence and increase in number of recent strains. The presence of three separate lineages of EV68 has been proposed, but not all available EV68 strains were used in that study [3]. We, however, performed more detailed analyses, adding new sequences from the UK from 2009 and 2010, and including all available EV68 sequences from the GenBank covering 5′NTR, VP4/VP2 as well as VP1.

In our study over two winters, about 10% of the respiratory entero/rhinovirus detection in respiratory samples using a commercial multiplex PCR test were enteroviruses and 50% of these respiratory enteroviruses were confirmed as EV68, which was detected in two consecutive winter season with no detection in the summer period between the two winters. The pattern of illness associated with EV68 was very similar to that of rhinoviruses and could be associated with severe respiratory tract infection. In our cohort, 8 patients required admission to intensive care unit. Compared to other entero/rhinovirus infection, EV68 was significantly associated with underlying chronic pulmonary diseases and asthma (Table 1).

Apart from a single strain from December 2009, EV68 circulating in South-East London in 2009 was different from that circulated in 2010. The differences between the strains from the two years were supported by phylogenetic analysis of sequences from the 5′NTR, VP4/VP2 and VP1 regions. The two viral lineages showed various degree of similarity with EV68 isolates from other years in other countries. Despite this difference, EV68 remains monophyletic as the sequence homology of global isolates over time remain >90% in all gene regions studied. However, contemporary EV68 strains diverge significantly from the original 1962 Fermon isolate, which showed 82.8%–89.3% nucleotide similarities in VP1 (88.1%–92.5% for amino acids).

A previous study based only on VP1 suggested that there were three separate lineages of EV68 [3]. However, the phylogenetic analysis in this study of global EV68 isolates suggested the presence of 2 modern lineages with one showing two sub-lineages and the presence of variants. We propose the following characteristics for the assignment to lineage 2:

A) Glycine deletion in position 141 in the VP1 protein;

B) Amino acid change at position 142 and 143 to threonine and glutamic acid in the VP2 protein;

and C) Presence of only one deletion block from 681–704 in the 5′NTR.

Nucleotide similarities cannot be used to characterise the lineages, sub-lineages and variants as the ranges of percentage homology overlap (84–93% similarities between lineages, 89–95% between sub-lineages and 96–98% between variants, Table 5). Worth mentioning is the difference in VP2, a surface protein on EV68. In the Fermon strain the amino acids serine and asparagine with polar uncharged side chains are in position 142 and 143. The side chains of alanine and glycine in lineage 1 are hydrophobic and neutral, whereas threonine and glutamic acid in lineage 2 are polar uncharged and negatively charged. These amino acid changes might result in different biological behaviour or even altered antigenicity.

Sub-lineages 1.1 and 1.2 possibly could be assigned on the basis of the nucleotide sequence in the islet region in the 5′NTR deletion. Limited data available from 2010 Yamagata isolates suggest a differentiation according to the nucleotide composition in the islet region (705–716) and one nucleotide difference in the lengths of both deletion block (681–703 and 717–728 for sub-lineage 1.1) (Figure S5). However, more sequences are needed for verification.

While the 2 lineages predominated in the UK in different years, co-circulation of lineages was observed in other countries. The relatedness between strains from different countries suggests that EV68 circulated rapidly between countries and continents. It is also possible that the prevalence of the EV68 strains in each country is dictated by the local herd immunity. A study in Finland has shown a high population seroprevalence of EV68 [17]. Hence, EV68 has been circulating in the community for many years. The lack of previous detection may be due to the presence of many transient and sub-clinical infections which were not tested. EV68 is an RNA virus with known potential of undergoing antigenic change. This study corroborates previous evidence that a rapid change is occurring in EV68, as reported for other human enteroviruses such as EV71 and echovirus 30 [18], [19], [20].

Recombination between the same species of enteroviruses is a common phenomenon [21], therefore effort should be made to monitor this possibility between EV68 and other species D enteroviruses such as EV70, EV94 and EV111. So far, no evidence of inter-typic recombination between 5′NTR and VP1 was observed from our limited sequence data and that in the literature (Figure S6). Only few studies of EV68 have investigated multiple genomic regions (Table S2) which greatly restricted the analysis of inter-typic and inter-lineage recombination. No bootstrap supported clustering between the lineages could be identified in the 5′NTR due to the high conservation in this region which prevented the analysis of inter-lineage recombination between 5′NTR and VP1. No evidence of inter-lineage recombination of strains was observed on analysis of available sequences covering VP4/VP2 and VP1 (n = 70). Apart from the 4 full genome sequences, only the American sequences (n = 15, 1963–2003) cover the 3D polymerase region. The reporting of more full viral genome sequences is required to help in the analysis of recombination.

This study was limited by the lack of sequence information on all target regions for each strain. The presence or absence of deletion blocks in the 5′NTR immediately preceding VP4 was not certain in most strains, as this region was not covered in most available sequences in the GenBank. Continuous surveillance and longer term study of sequences over several years is needed to establish the pattern of evolution and to understand the dynamics of transmission between countries and continents.

In conclusion, the recent increase in reports of EV68 could be due to improved surveillance and detection techniques, but may also be a real resurgence as a result of sequence variation interacting with population host immunity. This study suggested that modern EV68 strains consisted of two lineages and further sub-lineages; and that the global spread of EV68 has been occurring between and within continents in the past few years. Hence, EV68 is an emerging pathogen that requires global monitoring.

## Supporting Information

Figure S1 **Phylogenetic analysis of VP1 (uncollapsed).**
(TIF)

Figure S2 **Expansion of the phylogenetic analysis of VP1 for lineage 1.**
(TIF)

Figure S3 **Expansion of the phylogenetic analysis of VP1 for lineage 2.**
(TIF)

Figure S4 **Deletion of glycine in position 141 of the VP1 protein in lineage 2.**
(TIF)

Figure S5 **Deletion in the 5′NTR.**
(TIF)

Figure S6 **Phylogenetic analysis of group D enterovirus strains where both 5′NTR and VP1 sequences were available, showing the absence of inter-typic recombination.**
(TIF)

Table S1 **Reports of enterovirus 68 in the literature.**
(DOC)

Table S2 **Available EV68 sequences in GenBank.**
(DOCX)
