# Supplementary figures and images for: Lineages, Sub-Lineages and Variants of Enterovirus 68 in Recent Outbreaks
Source: PLoS One. 2012 Apr 20;7(4):e36005. doi: 10.1371/journal.pone.0036005 (PMC3335014; doi:10.1371/journal.pone.0036005)

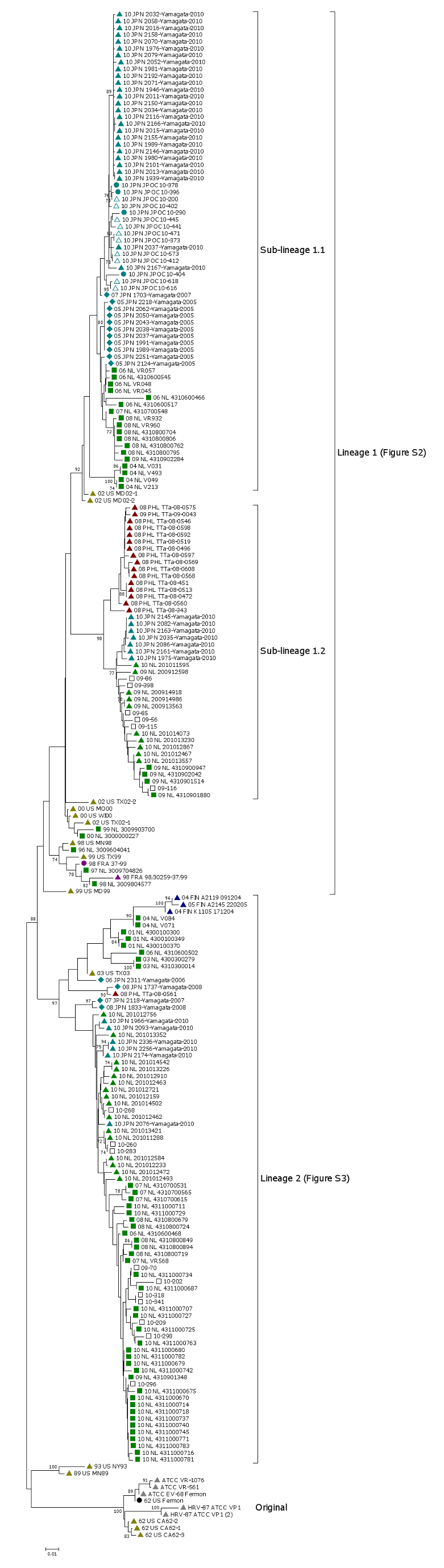

Supplement: Figure S1 — Phylogenetic analysis of VP1 (uncollapsed). (TIF) [file pone.0036005.s001.tif]

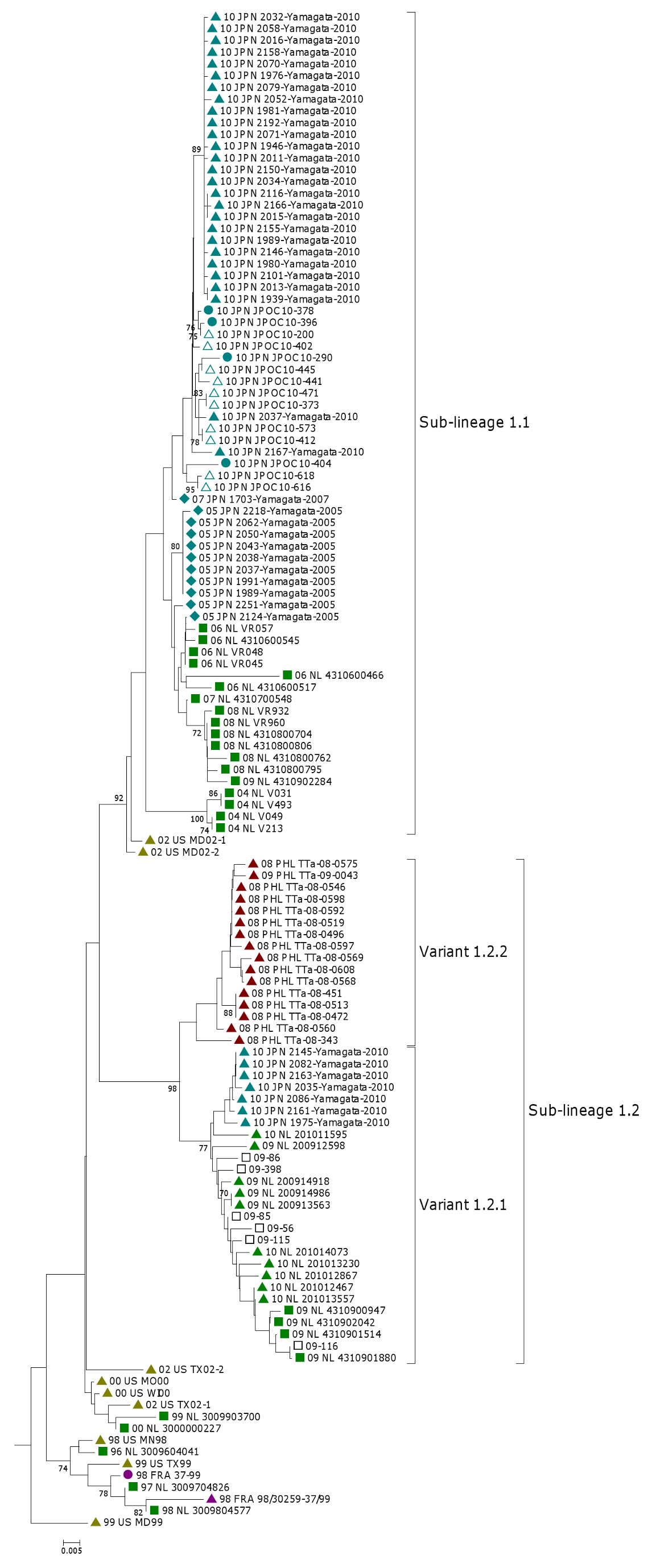

Supplement: Figure S2 — Expansion of the phylogenetic analysis of VP1 for lineage 1. (TIF) [file pone.0036005.s002.tif]

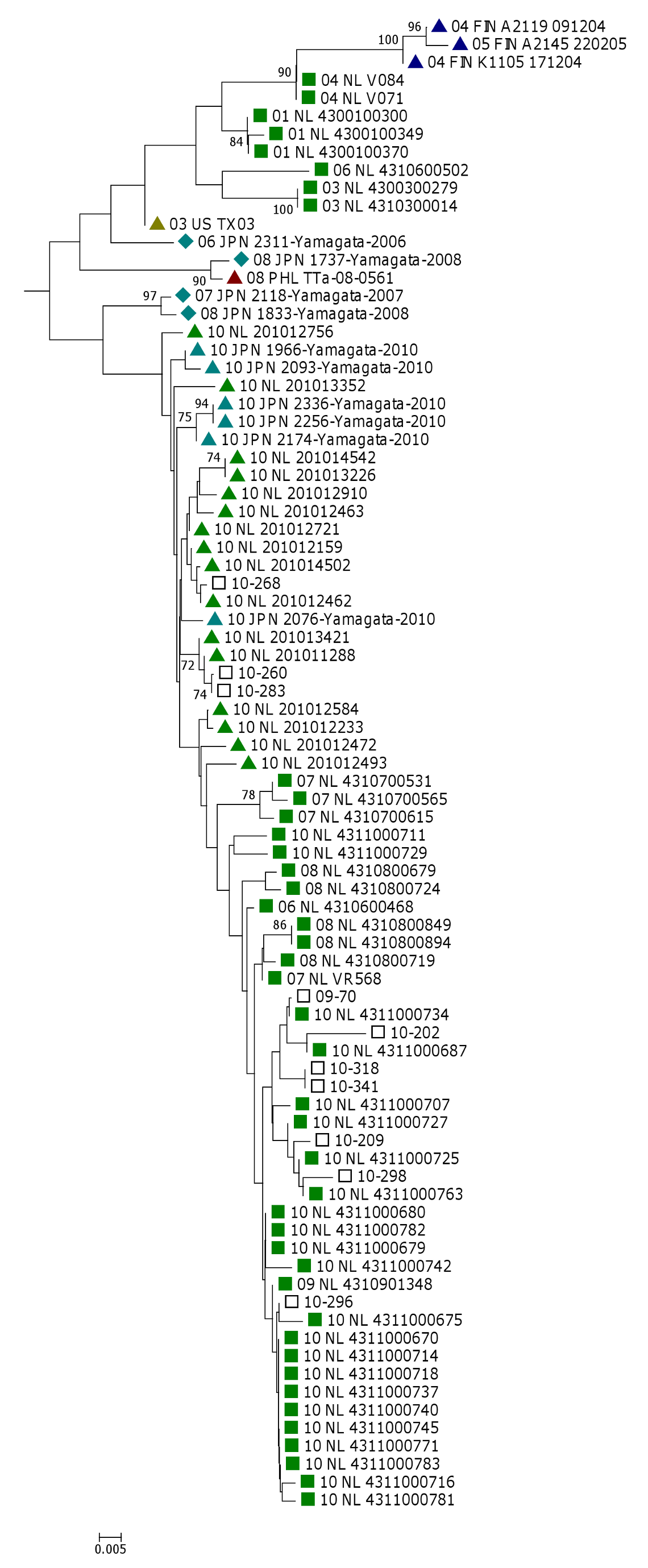

Supplement: Figure S3 — Expansion of the phylogenetic analysis of VP1 for lineage 2. (TIF) [file pone.0036005.s003.tif]

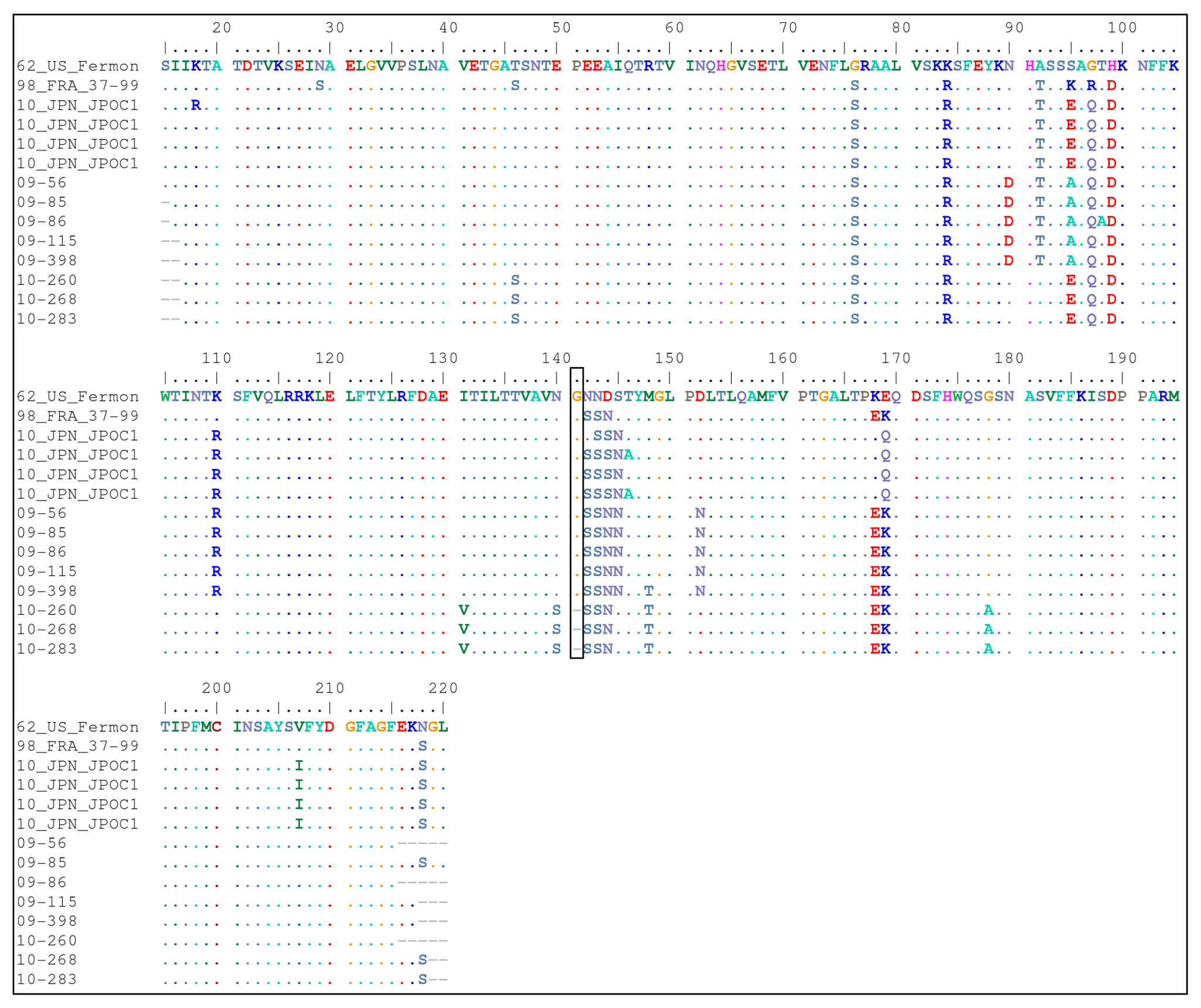

Supplement: Figure S4 — Deletion of glycine in position 141 of the VP1 protein in lineage 2. (TIF) [file pone.0036005.s004.tif]

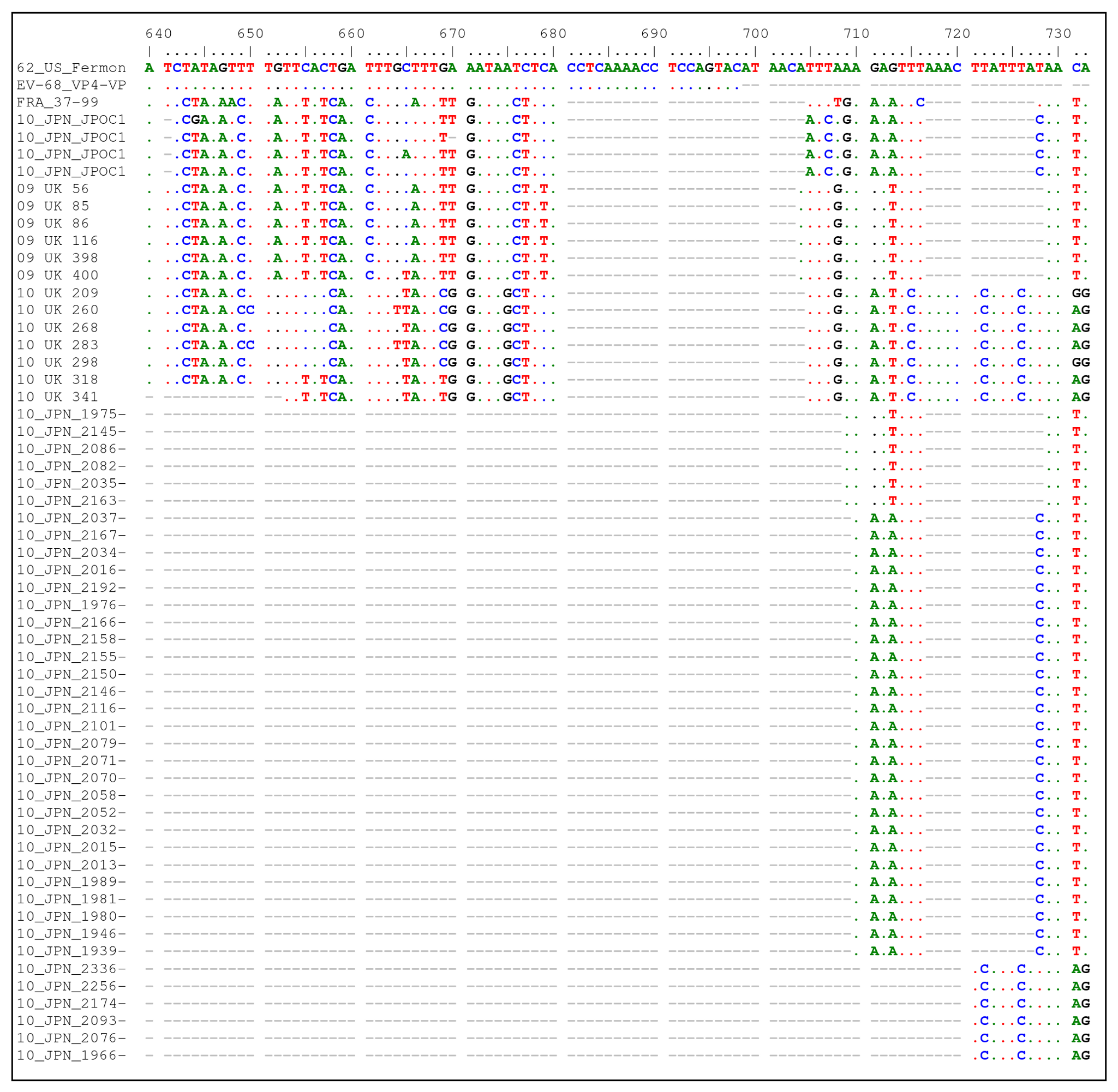

Supplement: Figure S5 — Deletion in the 5′NTR. (TIF) [file pone.0036005.s005.tif]

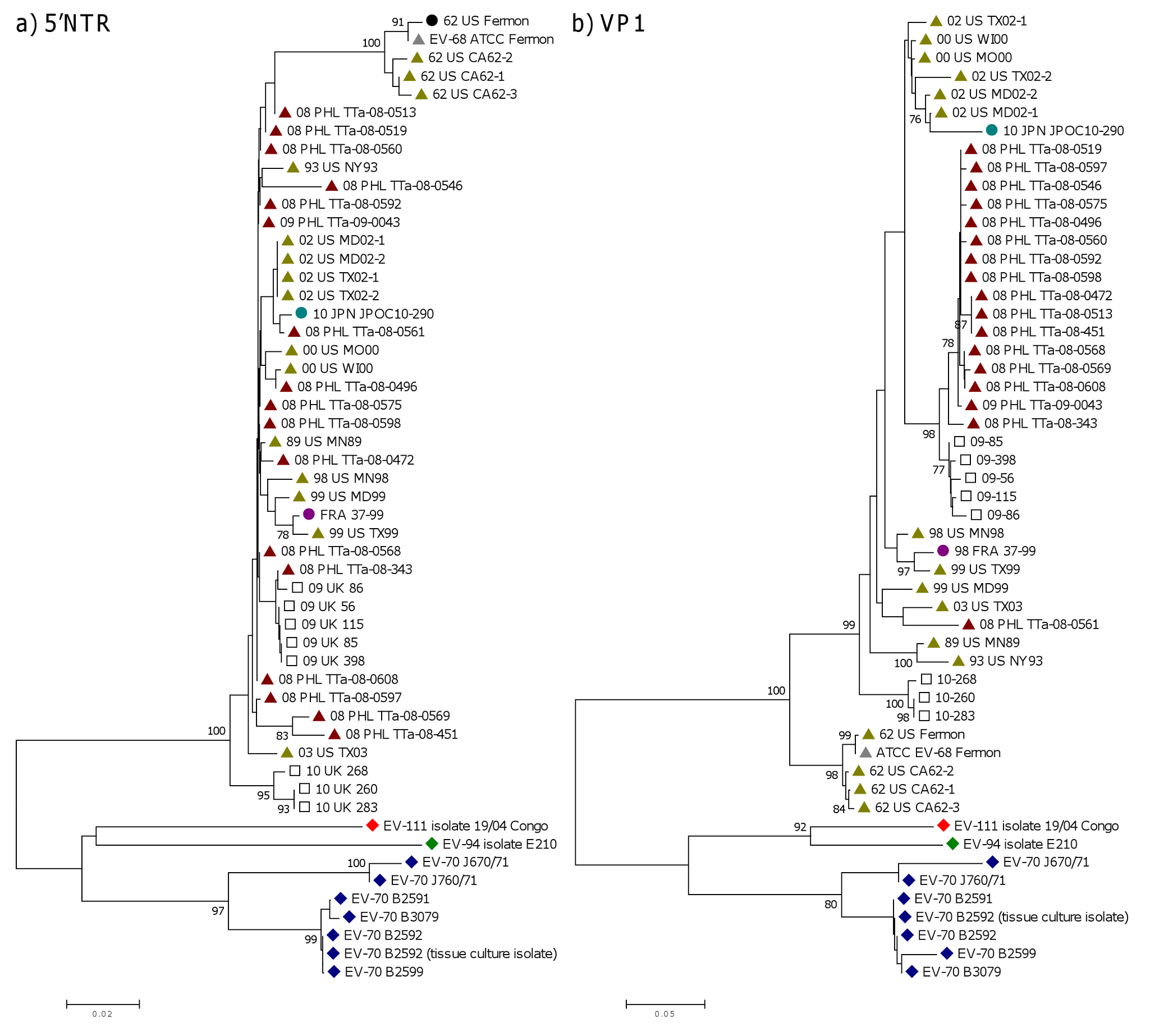

Supplement: Figure S6 — Phylogenetic analysis of group D enterovirus strains where both 5′NTR and VP1 sequences were available, showing the absence of inter-typic recombination. (TIF) [file pone.0036005.s006.tif]
