# Supplementary material for: Lineages, Sub-Lineages and Variants of Enterovirus 68 in Recent Outbreaks
Source: PLoS One. 2012 Apr 20;7(4):e36005. doi: 10.1371/journal.pone.0036005 (PMC3335014; doi:10.1371/journal.pone.0036005)
Supplement: Table S1 — Reports of enterovirus 68 in the literature. (DOC) [file pone.0036005.s007.doc]

**Table S1. Reports of enterovirus 68 in the literature.**

| **Location** | **Year** | **Specimen type** | **Total specimens** | **EV positive** | **EV 68** | **Note** | **Reference** |
| --- | --- | --- | --- | --- | --- | --- | --- |
| Berkeley, USA | 1962 | Throat swab | na | 4 | 4 | First report |  |
| Finland | 1983, 1993, 2002 | Serum | na | na | 100% | 100% seroprevalance in serum from pregnant women |  |
| New Hampshire, USA | Fall 2008 | CNS | na | na | 1 | Case report |  |
| Congo | na | Faecal sample | na | na | 1 | AFP patients |  |
| Surveillance USA | 1970 – 2005 | na | na | 52812 | 26 | First report in CNS (2005) |  |
| Surveillance | 2000 – 2007 | na | na | 1121 | 5 | Majority typed samples from Finland, rest untypable non-polio EV from WHO Polio Laboratory Network |  |
| Surveillance Japan^3^ | 2007 – 2011 | na | na | na | 144^1^ | 129 cases in 2010 with detailed information for only 11 cases | IDSC Infectious Agents Surveillance Centre* |
| Finland | 2004 – 2005 | Sputum | 386 | 36^2^ | 5 | Military recruits with and without history of asthma during respiratory infection |  |
| USA | 2004 – 2005 | Throat swabs | 97 | 8 | 7 | Military recruits with previous PCR positive HRV result |  |
| Commercial (USA) | 09/2005 – 12/2007 | Untypable isolates | 50 | 39 | 6 | Available from US company |  |
| Sao Paulo, Brazil | 09/2006 – 09/2007 | Nasopharyngeal aspirates or nasal mucus | 408 | 139^2,4^ | 4 | Children with cystic fibrosis during scheduled and unscheduled visits |  |
| Europe (GB, DE, BE, PL, NL SP) | Winter seasons 2007 – 2009 | Nasopharyngeal swabs | 482 | 6^2^ | 1 | HRV surveillance in adults with RTI and asymptomatic individuals | Poster (2011) ECCMID in Milan, Zlateva et al. (GRACE study group) |
| Caen, France | 2008 | Nasal sample | 327 | 68 | 19 |  |  |
| Eastern Philippines^3^ | 05/2008 – 05/2009 | Nasopharyngeal swabs | 816 | 29 | 21 | Hospitalised patients with severe pneumonia |  |
| Northern Italy | 10/2008 – 09/2009 | Respiratory samples | 1500 | 30^2^ | 12 | Children and adults with diagnosis of respiratory infection |  |
| New York City, USA^3^ | 05/2009 – 05/2010 | Nasopharyngeal swabs | 940 | 24 | 20 | Outpatients with fever or respiratory symptoms |  |
| Philadelphia, USA^3^ | Aug – Oct 2009 | na | na | 390^2,4^ | 28 |  |  |
| Atlanta, USA^3^ | 09/2009 – 04/2004 | na | 68 | na | 6 |  |  |
| Arizona, USA^3^ | Aug – Sep 2010 | Nasopharyngeal swabs | 7 | na | 5 | Upsurge in paediatric admissions for LRTI and despite viral testing and blood cultures no pathogen was detected |  |
| Osaka, Japan | Jun – Oct 2010 | Respiratory samples | 448 | 31^2^ | 14 | Patients with RTI and fevers |  |
| Japan | Jul – Sep 2010 | Nasopharyngeal samples | 35 | na | 26 | Children admitted to hospital with a history of asthma |  |
| Northern Netherlands^3^ | 06/2009 – 01/2011 | Mainly nasopharyngeal swabs or aspirates | 272 | 24 | 24 | Children and adults |  |
| Netherlands | 1994 – 2010 | Nose and throat swabs | 13310 | 317 | 71 | Retrospective analysis |  |

^1^As reported by 23.01.2012

^2^not all EV positive specimens sequenced

^3^CDC report

^4^Entero/rhinovirus or only rhinovirus detection

* <https://hasseidoko.mhlw.go.jp/Byogentai/Pdf/data60e.pdf>
