# Supplementary material for: Lineages, Sub-Lineages and Variants of Enterovirus 68 in Recent Outbreaks
Source: PLoS One. 2012 Apr 20;7(4):e36005. doi: 10.1371/journal.pone.0036005 (PMC3335014; doi:10.1371/journal.pone.0036005)
Supplement: Table S2 — Available EV68 sequences in GenBank. (DOCX) [file pone.0036005.s008.docx]

**Table S2. Available EV68 sequences in GenBank.**

| **Country** | **Year(s)** | **Name** | **Number** | **Region** | **Symbol in phylogenetic tree** | **Published** | **GenBank** |
| --- | --- | --- | --- | --- | --- | --- | --- |
| US | 1962 | Fermon | 1 | Full genome |  | Yes | AY426531 |
| France | 1998 | 39-99 France | 1 | Full genome |  | Yes | EF107098 |
| France | 1998 | 98/30259-37/99 | 1 | VP1 |  | Yes | AY208116 |
| Japan, Osaka | 2010 | JPOC10-* | 2 | Full genome |  | Yes | AB601882-3 |
|  |  |  | 2 | Coding region |  | Yes | AB601884-5 |
|  |  |  | 10 | VP4 |  | Yes | AB601862-71 |
|  |  |  | 10 | VP1 |  | Yes | AB601872-81 |
| Brazil | 2007 | VFC-* | 4 | 5’NTR |  | Yes | GU933068-70, -77 |
| Philippines | 2008 – 2009 | TTa-* | 21 | 5’NTR |  | Yes | AB569257-77 |
|  |  |  | 17 | VP1 |  | Yes | AB569278-94 |
| USA | 1962 – 2003 | (individual) | 15 | 5’NTR |  | Yes | AY426516-30 |
|  |  |  | 15 | VP1 |  | Yes | AY426486-500 |
| Switzerland | 2008 | F91-* | 2 | VP4/VP2 |  | Yes | JF285167-68 |
| Japan, Yamaguchi | 2010 | Yamaguchi/Yu/* | 23 | VP4/VP2 |  | Yes | AB618506-28 |
| Italy | 2008 – 2009 | Pav* | 13 | VP4/VP2 |  | Yes | HM370281-93 |
| Japan, Yamagata | 2010 | *-Yamagata-2010 | 39 | VP4/VP2 |  | No | AB612278-316 |
|  |  |  | 40 | VP1 |  | No | AB614406-45 |
| Finland | 2004 | (individual) | 3 | VP1 |  | Yes | GQ466485-7 |
| Japan, Yamagata | 2005 – 2008 | *-Yamagata-* | 15 | VP1 |  | No | AB667885-99 |
| Netherlands | 2009 – 2010 | EV68_NL_* | 26 | VP1 |  | Yes | JF896287-312 |
| Netherlands | 1994 – 2010 | (individual) | 69 | VP1 |  | Yes | JF830017-85 |
| ATCC | NA | (individual) | 6 | 5’NTR |  | NA | ^1^ |
|  |  |  | 6 | VP4 |  | NA | ^2^ |
|  |  |  | 5 | VP1 |  | NA | ^3^ |

* replaced by unique identifier for sequence

^1^AY062274, AY062275, AY062273, AF412375, EU870491, EU870464

^2^AY040243, AY062276, AY062277, X87604, AB079223, AB061487

^3^AY355268, AY062280, AY062278, AY062279, AF081348
